# Region-specific variation and determinants of caesarean delivery among ever-married women in Bangladesh

**DOI:** 10.1371/journal.pone.0328830

**Published:** 2025-09-12

**Authors:** Gobinda Karmakar, Md. Tariqujjaman, Aklima Alam, Md Ahshanul Haque, Nurun Nahar Naila, Tahmeed Ahmed

**Affiliations:** Nutrition Research Division, icddr, b, Dhaka, Bangladesh; Australian National University Faculty of Arts: Australian National University College of Arts and Social Sciences, AUSTRALIA

## Abstract

**Background:**

The extensive use of caesarean delivery has adverse consequences for both maternal and neonatal health. This study aims is to investigate regional variation and identify region-specific determinants of caesarean deliveries among ever-married women in Bangladesh.

**Methods:**

A total of 17,704 women aged 15–49 who had given birth to children within three years preceding each of the surveys were included in this study, based on the last four consecutive nationally representative surveys between 2011 and 2022. Various demographics and socio-economic variables were considered as exposure variables. Bivariate and multiple mixed-effects logistic regression models were used to draw inferences from the data.

**Results:**

In Bangladesh, between 2011 and 2022, the percentage of caesarean deliveries increased threefold (15% to 46%). Additionally, more than half of the children born in Dhaka and Khulna were via caesarean deliveries. After adjusting for relevant covariates in each of the region-specific models, women with highly educated husbands had significantly higher odds of undergoing a caesarean delivery compared to those with less educated husbands in Barisal (OR: 4.48), Chattogram (OR: 2.69), and Rangpur (OR: 2.46) divisions. The likelihood of caesarean delivery was considerably higher among overweight or obese women in Dhaka and Sylhet (OR: 2.33 and 2.50), as well as among women living in households with higher wealth status than their counterparts in Sylhet and Khulna (OR: 2.95 and 2.22), respectively.

**Conclusion:**

Policymakers can address the high rate of caesarean deliveries by targeting several key factors at various geographic levels. Raising family awareness about the benefits of normal delivery can encourage expectant parents to make informed choices. They should also ensure the quality of care provided in hospitals, ensuring that medical professionals follow evidence-based guidelines for childbirth. Furthermore, implementing a centralized or local pregnancy registration system may enhance the monitoring and access to maternal health services.

## Introduction

Approximately 18.5 million caesarean deliveries are performed globally each year [1], and the number is expected to increase in middle- and high-income countries [2]. The global rate of caesarean deliveries increased by 6 percentage points from 2010 to 2018, whereas in 2018, the Latin American and Caribbean countries contributed almost 43%, in contrast with the Sub-Saharan African regions contributing only 5%. From 1990 to 2018, the global rate of caesarean deliveries rose from 6.7% to 21.1% [3].

The extensive and unrestricted use of caesarean delivery varies according to socio-demographic indicators and regional factors [4,5] and it also has adverse consequences for maternal and neonatal outcomes [6]. Despite significant disparities at different geographical regions, global caesarean delivery rates have been steadily increasing [7]. Twenty-one percent of women in the world gave birth to their children by caesarean delivery [8], exceeding the World Health Organization (WHO) recommended range of 10% to 15% at the population level [9]. In Bangladesh, the caesarean delivery rate increased by 28 percentage points from 2011 to 2022 (17% in 2011 to 45% in 2022) [10,11]. The caesarean delivery rates at different geographical locations were at least double in 2022, as compared to 2011, with Dhaka and Khulna reporting much higher rates of change, 53% and 66%, respectively. In 2011, the caesarean rate was roughly 10% of all births everywhere apart from in Khulna, where it was 26% [11]. This rising caesarean delivery rate is certainly a major public health concern for Bangladesh [10].

An excessive number of caesarean deliveries are performed without adequate justification or clear medical indications such as fetal distress, non-cephalic presentation, maternal disease, cephalopelvic disproportion, inadequate labor progression, multiple pregnancies, etc. [12–14]. A study estimated that in 2008, around 6.2 million unnecessary caesarean deliveries were performed, costing over US$2.32 billion [1,15]. Financial incentives to perform surgical deliveries may be a possible factor in the increasing rate of unnecessary caesarean delivery worldwide [15].

However, various non-medical factors such as low socioeconomic status, communication barriers, and lack of support during labor and delivery have also been significantly associated with the increasing rate of caesarean delivery [16]. Numerous studies conducted in Bangladesh have identified several socioeconomic and demographic characteristics linked to caesarean delivery, including maternal education, place of residence, wealth index, maternal age, age at first birth, parity, birth order, antenatal visits, husband’s occupation, delivery in the private sector, religion, and divisions [4,17–19]. As previous studies have shown, several individual characteristics, including sociodemographic, health, medical, and institutional factors, can contribute to the rate of caesarean deliveries [20].

Healthcare provider retention is a serious issue in Bangladesh, especially in remote areas, leading to many vacancies in public health facilities [21]. However, the Khulna and Dhaka divisions have comparatively fewer vacant positions, but a relatively high number of accessible emergency obstetric care facilities in the public sector in the Khulna region, resulting in the higher usage of maternity care services in these administrative regions [22]. The higher rate of caesarean delivery in Khulna, despite Dhaka’s superior healthcare infrastructure, can be attributed to several regional factors like antenatal care coverage, maternal health services [23]. Currently, there is a gap in the literature regarding the prevalence and contributing factors of caesarean deliveries across different geographic locations in Bangladesh, presenting a promising opportunity for further investigation. The goal of this study is to explore region-specific variations and identify the region-specific determinants of caesarean deliveries among ever-married women aged 15–49 years in Bangladesh.

## Methods

### Data source

This analysis included data from four waves of the Bangladesh Demographic and Health Survey (BDHS), conducted in 2011, 2014, 2017−18, and 2022. Before 2011, Bangladesh consisted of only six administrative divisions, which led to the exclusion of pre-2011 surveys from our analysis. However, Mymensingh was not an administrative division until 2018 and was previously part of the Dhaka Division in earlier surveys. To maintain consistency with the BDHS data from 2011 and 2014, we merged the Mymensingh Division with the Dhaka Division for the survey years 2018 and 2022. We included a total of 17,704 ever-married women aged 15–49 years who had given birth to children three years preceding each survey. These participants were drawn from four rounds of nationally representative surveys conducted in Bangladesh. Particularly, 5,554 women were included from the 2011 survey, 4,466 from the 2014 survey, 5,042 from the 2017–18 survey, and 2,643 from the most recent survey conducted in 2022. These cross-sectional household surveys, conducted in all seven administrative regions (divisions) of Bangladesh Barisal, Chattogram, Dhaka, Khulna, Rajshahi, Rangpur, and Sylhet covered both rural and urban areas and were nationally representative. Each division is subdivided into districts, and each district is divided into sub-districts (Upazilas), which are further divided into rural and urban areas. In sub-districts (Upazilas), urban areas are divided into wards, which are further subdivided into mohallas, and rural areas are divided into union parishads (UPs), and within each UP, there are mouzas. These divisions help distinguish between rural and urban areas across the country.

### Sampling procedure

The BDHS surveys employed a two-stage stratified sampling procedure. In the first stage of sampling, 600 from the 2011 and 2014 surveys and 675 from the 2017−18 and 2022 surveys primary sampling units were chosen at random using the sampling frame provided by the Bangladesh Bureau of Statistics, with the probability of selection inversely related to the size of the unit. In the second stage of sampling, 30 households from the 2011, 2014, and 2017−18 surveys and 45 households from the 2022 survey were selected by systematic random sampling within each primary sampling unit. Detailed information regarding the sampling strategy and other BDHS-related matters can be obtained elsewhere [4,17,24].

### Outcome variable

A variety of childbirth data, including infant weight, delivery method, and pregnancy complications, were collected for the BDHS from each of the respondents aged between 15–49 years. The question that was asked of participants was whether the baby was delivered by caesarean delivery, and the response was 0 “no” and 1 “yes”. The questions were asked of respondents who gave birth within the last three years. Hence, in our study, the outcome variable for caesarean section delivery was categorized as yes and no.

### Exposure variables

**Fig 1:**
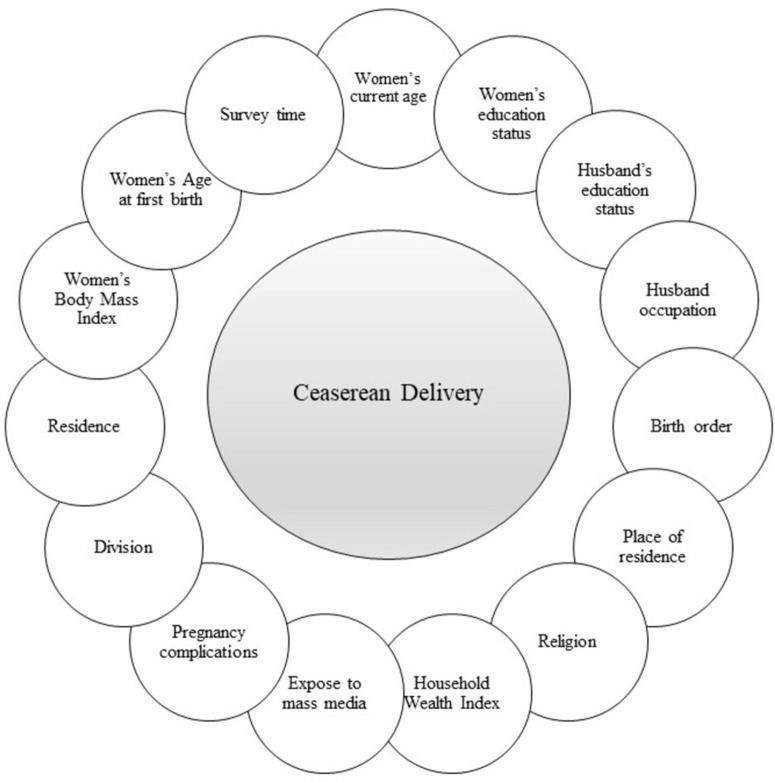
Conceptual framework of linkage of exposure variables and outcome variables.

This study utilized various sociodemographic variables, expanding on prior research that has highlighted the significance of these factors. The potential determinants used in the present study according to the prior literature were administrative divisions (Barisal, Chattogram, Dhaka, Khulna, Rajshahi, Rangpur, and Sylhet), women’s current age (15−19 years, 20−34 years, and 35−49 years), women’s educational status (No formal education or primary, secondary, and higher), age at first birth (<18 years, ≥ 18 years), husband’s educational status (No formal education or primary, secondary, and higher), husband’s occupation (agriculture, non-agriculture, others), place of residence (urban, rural), household wealth index (poor, middle, rich), women’s exposed to mass media (“yes” if the frequency of reading newspapers and/or magazines, listening to the radio, and watching television was less than once or at least once a week and otherwise “no”), birth order (1, 2, and 3 or more), maternal body mass index (underweight, normal, and overweight or obese), pregnancy complications (yes, no), survey time (2011, 2014, 2017−18 and 2022). Fig 1 displays the conceptual framework of the linkage of exposure and outcome variables.

### Ethical statement

The BDHS surveys were conducted under the authority of the National Institute of Population Research and Training (NIPORT) of the Ministry of Health and Family Welfare. Mitra and Associates, a Bangladeshi research firm, implemented the survey with technical assistance provided by ICF International as part of its Demographic and Health Survey Programs (MEASURE DHS). The survey methodology and questionnaire were reviewed and approved by the Institutional Review Board of ICF, and written consent was obtained from respondents before the interviews. Consequently, the author did not have access to the original documents related to ethical approval and consent forms.

### Statistical analysis

All the analyses were performed using Stata (version 15.0; Stata Corp., 4905 Lakeway Drive, College Station, Texas 77845, USA) and R (version 4.4.2). To achieve the objectives of this study, both descriptive and inferential analyses were conducted. For descriptive analysis, weighted frequencies with proportions and weighted means with standard deviations (SD) were used to summarize the data. The chi-square test, as well as a simple mixed-effects logistic regression model, was used to assess the bivariate association between the caesarean delivery and all related covariates. To estimate the strength of these associations, multiple mixed-effects logistic regression models were performed using R, treating the primary sampling unit as a cluster, and adjusting for relevant covariates and cluster-level variations to compare region-specific differences. Using the pooled data, a mixed model was also performed to explore the combined effect of “survey time” and “divisions” along with the covariates to observe the true effect on caesarean delivery. We assessed the Akaike Information Criterion (AIC) and the Bayesian Information Criterion (BIC) for each of the models. Also, we calculated the area under the Receiver Operating Characteristic (ROC) curve to evaluate model performance [25–27]. The prevalence of caesarean delivery has been mapped to show regional (divisional) variation. The maps were created in R using the administrative shapefile available at the Humanitarian Data Exchange https://data.humdata.org/dataset/cod-ab-bgd website. The dataset, sourced from the Bangladesh Bureau of Statistics (BBS) via the OCHA Regional Office for Asia and the Pacific (ROAP), is licensed under CC BY 4.0 (https://creativecommons.org/licenses/by/4.0/).

## Results

**Fig 2:**
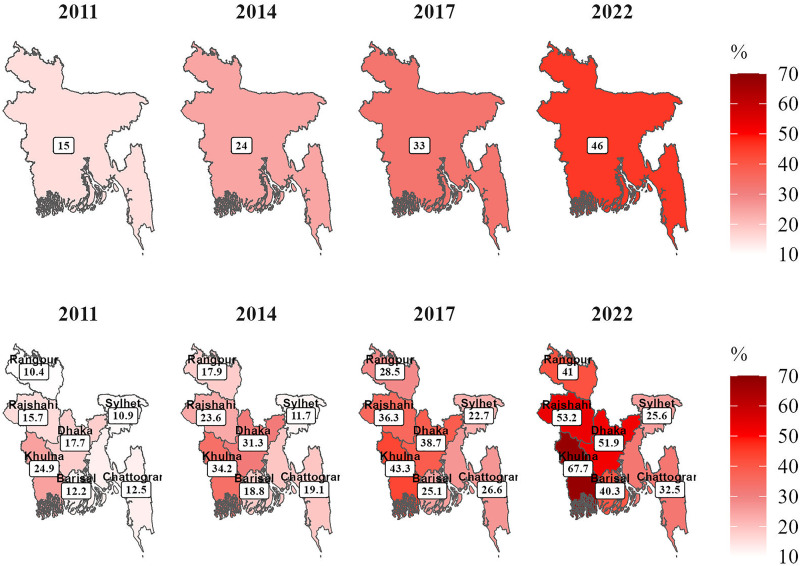
Region-specific variations of caesarean delivery among ever-married women aged 15–49 years in Bangladesh from 2011 to 2022. **The divisional level shapefiles of Bangladesh were obtained from the open-source website Humanitarian Data Exchange (** **https://data.humdata.org/dataset/cod-ab-bgd** **) and are licensed under CC BY 4.0. This figure is a replacement for previously copyrighted maps and has been recreated using openly licensed data for illustrative purposes.**

The background characteristics of study participants are described in Table 1. A total of 17,704 ever-married women aged 15–49 were included in this study, drawn from the four latest consecutive nationally representative surveys. The proportion of ever-married women who had caesarean births was 15%, 24%, 33%, and 46% in the survey years 2011, 2014, 2017−18, and 2022, respectively (Fig 2), and it varied by demographic, socio-economic, and geographical characteristics (Table 2). More than forty percent of women gave birth to their first child before the age of eighteen, and more than seventy percent of all women were between the ages of 20 and 34. More than half of the participants had completed their secondary or higher educations. In all regions, the proportion of women between the ages of 15 and 49 who were regularly exposed to mass media exceeded 61.8%. Overall, 36% of the mothers experienced difficulties during their pregnancy to give birth to their child, whereas the proportions were 40% and 42% in Khulna and Rangpur divisions, respectively. Around 19% of mothers were found to be overweight or obese in the Chattogram and Dhaka divisions, while 20% of mothers were underweight in the Khulna division.

**Table 1:** Background characteristics of the study participants.

Characteristic	Barisal	Chattogram	Dhaka	Khulna	Rajshahi	Rangpur	Sylhet	Overall
	N = 995	N = 4,023	N = 5,841	N = 1,563	N = 1,995	N = 1,839	N = 1,449	N = 17,704
**Women’s current age (in years), n (%)**								
15–19 years	181 (18.23)	667 (16.57)	1,032 (17.67)	338 (21.66)	385 (19.28)	436 (23.70)	161 (11.10)	3,200 (18.07)
20–34 years	755 (75.87)	3,116 (77.45)	4,452 (76.23)	1,144 (73.20)	1,507 (75.51)	1,306 (71.04)	1,168 (80.62)	13,448 (75.96)
35–49 years	59 (5.91)	240 (5.98)	356 (6.09)	80 (5.14)	104 (5.21)	97 (5.27)	120 (8.28)	1,056 (5.97)
**Women’s educational status, n (%)**								
No formal education or primary	376 (37.75)	1,468 (36.49)	2,525 (43.23)	418 (26.72)	782 (39.18)	688 (37.41)	833 (57.46)	7,089 (40.04)
Secondary	475 (47.77)	2,142 (53.26)	2,524 (43.22)	924 (59.16)	951 (47.64)	911 (49.52)	525 (36.24)	8,453 (47.74)
Higher	144 (14.48)	412 (10.25)	791 (13.55)	221 (14.12)	263 (13.17)	240 (13.07)	91 (6.30)	2,163 (12.22)
**Women’s age at first birth, n (%)**								
< 18 years	436 (43.79)	1,684 (41.86)	2,412 (41.30)	739 (47.29)	992 (49.71)	986 (53.60)	491 (33.88)	7,739 (43.71)
≥ 18 years	559 (56.21)	2,339 (58.14)	3,429 (58.70)	824 (52.71)	1,003 (50.29)	853 (46.40)	958 (66.12)	9,965 (56.29)
**Husband’s educational status, n (%)**								
No formal education or primary	499 (50.12)	2,006 (49.86)	3,060 (52.40)	679 (43.42)	1,102 (55.24)	1,013 (55.06)	976 (67.38)	9,334 (52.72)
Secondary	346 (34.76)	1,468 (36.48)	1,790 (30.64)	609 (38.95)	554 (27.78)	527 (28.66)	348 (24.04)	5,642 (31.87)
Higher	150 (15.11)	549 (13.66)	991 (16.96)	275 (17.62)	339 (16.98)	299 (16.28)	124 (8.58)	2,728 (15.41)
**Husband’s occupation, n (%)**								
Non-agriculture	825 (82.88)	3,314 (82.39)	4,679 (80.11)	1,128 (72.19)	1,328 (66.57)	1,134 (61.66)	1,057 (72.95)	13,465 (76.06)
Agriculture	147 (14.79)	578 (14.37)	996 (17.05)	396 (25.36)	629 (31.51)	672 (36.52)	332 (22.90)	3,750 (21.18)
Others	23 (2.33)	130 (3.24)	166 (2.84)	38 (2.45)	38 (1.92)	33 (1.82)	60 (4.16)	489 (2.76)
**Place of residence, n (%)**								
Urban	175 (17.63)	926 (23.03)	2,133 (36.51)	359 (22.99)	349 (17.47)	239 (13.01)	197 (13.62)	4,379 (24.73)
Rural	820 (82.37)	3,096 (76.97)	3,708 (63.49)	1,203 (77.01)	1,647 (82.53)	1,600 (86.99)	1,252 (86.38)	13,326 (75.27)
**Religion**								
Muslim	927 (93.18)	3,667 (91.15)	5,567 (95.32)	1,365 (87.35)	1,893 (94.84)	1,559 (84.80)	1,274 (87.94)	16,253 (91.80)
Others	68 (6.82)	356 (8.85)	273 (4.68)	198 (12.65)	103 (5.16)	280 (15.20)	175 (12.06)	1,452 (8.20)
**Household wealth index, n (%)**								
Poor	568 (57.11)	1,465 (36.42)	2,049 (35.09)	578 (36.98)	936 (46.90)	1,140 (62.00)	730 (50.39)	7,467 (42.18)
Middle	201 (20.17)	876 (21.77)	1,036 (17.73)	355 (22.72)	459 (22.98)	298 (16.19)	234 (16.18)	3,458 (19.53)
Rich	226 (22.72)	1,682 (41.81)	2,755 (47.18)	630 (40.30)	601 (30.11)	401 (21.81)	484 (33.43)	6,779 (38.29)
**Exposed to mass media, n (%)**	457 (45.91)	2,440 (60.65)	3,953 (67.69)	1,070 (68.50)	1,345 (67.39)	1,040 (56.56)	636 (43.87)	10,941 (61.80)
**Birth order, n (%)**								
1	401 (40.27)	1,449 (36.03)	2,346 (40.16)	666 (42.62)	805 (40.36)	740 (40.22)	465 (32.08)	6,871 (38.81)
2	309 (31.05)	1,154 (28.69)	1,782 (30.52)	580 (37.10)	680 (34.06)	599 (32.57)	363 (25.09)	5,467 (30.88)
3+	285 (28.68)	1,419 (35.28)	1,713 (29.32)	317 (20.29)	510 (25.58)	500 (27.21)	621 (42.83)	5,366 (30.31)
**Maternal body mass index (BMI < 18.5), n (%)**								
Underweight	241 (24.26)	771 (19.16)	1,228 (21.02)	297 (18.98)	501 (25.10)	463 (25.19)	434 (29.94)	3,934 (22.22)
Normal	594 (59.72)	2,474 (61.49)	3,479 (59.57)	946 (60.55)	1,182 (59.26)	1,143 (62.16)	851 (58.76)	10,670 (60.27)
Overweight or obese	159 (16.03)	778 (19.34)	1,134 (19.41)	320 (20.47)	312 (15.65)	233 (12.65)	164 (11.30)	3,100 (17.51)
**Pregnancy complications, n (%)**	391 (39.34)	1,277 (31.75)	2,195 (37.58)	636 (40.67)	718 (36.01)	778 (42.31)	378 (26.10)	6,374 (36.00)
**Survey time, n (%)**								
2011	279 (28.03)	1,388 (34.51)	1,669 (28.58)	490 (31.38)	686 (34.40)	574 (31.23)	466 (32.16)	5,554 (31.37)
2014	263 (26.39)	980 (24.37)	1,579 (27.04)	359 (22.98)	445 (22.32)	440 (23.95)	399 (27.52)	4,466 (25.22)
2017	288 (28.91)	1,072 (26.65)	1,695 (29.03)	456 (29.20)	590 (29.55)	538 (29.26)	403 (27.83)	5,042 (28.48)
2022	166 (16.68)	582 (14.48)	897 (15.35)	257 (16.44)	274 (13.73)	286 (15.56)	181 (12.49)	2,643 (14.93)

BMI: Body mass index.

**Table 2:** Region-specific variations of caesarean delivery by background characteristics among the ever-married women aged 15-49 years in Bangladesh from 2011 to 2022.

Characteristic	Barisal	Chattogram	Dhaka	Khulna	Rajshahi	Rangpur	Sylhet
**Women’s current age (in years), n (%)**							
15–19 years	32 (17.65)	135 (20.19)	301 (29.12)	111 (32.75)	107 (27.78)	78 (17.98)	14 (8.87)
20–34 years	177 (23.40)	664 (21.31)	1,501 (33.72)	470 (41.09)	431 (28.59)	299 (22.87)	206 (17.65)
35–49 years	13 (22.95)	37 (15.40)	110 (31.04)	36 (44.67)	35 (33.78)	32 (32.97)	15 (12.42)
**Women’s educational status, n (%)**							
No formal education or primary	33 (8.86)	119 (8.11)	406 (16.09)	94 (22.53)	103 (13.13)	75 (10.87)	58 (6.97)
Secondary	106 (22.28)	527 (24.59)	981 (38.86)	367 (39.68)	295 (31.08)	197 (21.63)	127 (24.17)
Higher	83 (57.56)	190 (46.04)	525 (66.40)	156 (70.63)	175 (66.49)	137 (57.12)	50 (55.29)
**Women’s age at first birth, n (%)**							
< 18 years	59 (13.49)	247 (14.66)	537 (22.26)	219 (29.64)	195 (19.63)	125 (12.71)	35 (7.14)
≥ 18 years	163 (29.21)	589 (25.17)	1,375 (40.11)	398 (48.29)	378 (37.68)	284 (33.26)	200 (20.91)
**Husband’s educational status, n (%)**							
No formal education or primary	42 (8.34)	218 (10.88)	578 (18.88)	177 (26.13)	203 (18.43)	116 (11.42)	90 (9.25)
Secondary	93 (26.98)	365 (24.88)	699 (39.04)	257 (42.28)	170 (30.65)	137 (25.92)	93 (26.65)
Higher	87 (57.97)	252 (45.94)	636 (64.20)	182 (66.12)	200 (58.96)	157 (52.39)	52 (42.08)
**Husband’s occupation, n (%)**							
Non-agriculture	202 (24.51)	770 (23.22)	1,723 (36.83)	467 (41.41)	436 (32.85)	312 (27.51)	193 (18.27)
Agriculture	14 (9.64)	41 (7.07)	124 (12.47)	130 (32.83)	131 (20.87)	87 (12.89)	31 (9.30)
Others	6 (24.99)	25 (19.41)	65 (39.26)	20 (51.09)	5 (13.92)	11 (31.62)	11 (18.94)
**Place of residence, n (%)**							
Urban	71 (40.31)	241 (26.00)	991 (46.47)	169 (47.08)	153 (43.84)	88 (36.78)	53 (26.89)
Rural	151 (18.47)	595 (19.21)	921 (24.85)	448 (37.20)	420 (25.50)	321 (20.07)	182 (14.57)
**Religion, n (%)**							
Muslim	195 (21.01)	753 (20.52)	1,759 (31.60)	543 (39.77)	539 (28.49)	342 (21.92)	199 (15.59)
Others	27 (40.22)	83 (23.35)	153 (56.00)	74 (37.39)	34 (32.78)	67 (24.08)	37 (21.05)
**Household wealth index, n (%)**							
Poor	68 (12.05)	128 (8.74)	285 (13.92)	135 (23.35)	164 (17.55)	160 (14.06)	38 (5.14)
Middle	45 (22.20)	167 (19.07)	262 (25.33)	146 (41.08)	131 (28.65)	82 (27.63)	31 (13.16)
Rich	109 (48.26)	541 (32.15)	1,365 (49.53)	336 (53.35)	277 (46.14)	167 (41.52)	167 (34.48)
**Exposed to mass media, n (%)**	150 (32.75)	646 (26.48)	1,624 (41.07)	469 (43.79)	460 (34.21)	295 (28.40)	162 (25.47)
**Birth order, n (%)**							
1	110 (27.54)	392 (27.07)	947 (40.38)	299 (44.97)	309 (38.41)	204 (27.62)	109 (23.40)
2	77 (24.76)	242 (20.99)	629 (35.29)	238 (41.08)	193 (28.47)	141 (23.50)	75 (20.55)
3+	35 (12.36)	201 (14.17)	336 (19.64)	79 (24.97)	70 (13.72)	64 (12.80)	52 (8.37)
**Maternal body mass index (BMI < 18.5), n (%)**							
Underweight	25 (10.42)	105 (13.66)	191 (15.58)	82 (27.67)	99 (19.80)	67 (14.54)	31 (7.15)
Normal	129 (21.64)	445 (17.98)	1,039 (29.86)	346 (36.59)	323 (27.28)	243 (21.23)	135 (15.91)
Overweight or Obese	68 (42.90)	286 (36.72)	682 (60.16)	188 (58.91)	151 (48.41)	99 (42.58)	69 (42.12)
**Pregnancy complications, n (%)**	118 (30.13)	391 (30.62)	986 (44.91)	274 (43.14)	264 (36.72)	219 (28.16)	110 (29.20)

BMI: Body mass index.

### Region-specific variation of caesarean delivery

**Fig 2** displays the regional variation in the rate of caesarean delivery among ever-married women aged 15–49 years in Bangladesh from 2011 to 2022. The percentage of women who gave birth to children by caesarean deliveries went up from 15% in 2011 to 46% in 2022. This variation was also observed in the demographic, socioeconomic, and geographical characteristics of the participants (Table 2). In 2011, the prevalence of caesarean delivery in Dhaka, Chattogram, Rajshahi, Rangpur, and Sylhet was between ten to twenty percent, whereas it was more than twenty percent in Khulna. Compared to 2011, the rates of caesarean deliveries in every specific region increased by more than two to four times in 2022 in Bangladesh.

### Region-specific determinants of caesarean delivery

In the multiple regression analysis, women’s age, women’s educational status, age at first birth, husband’s educational status, husband’s occupation, place of residence, religion, wealth status, exposure to mass media, birth order, maternal body mass index, pregnancy complications, and survey time were the significant covariates for caesarean delivery. After adjusting for theses relevant covariates in each of the models across all seven regions, women with highly educated husbands had significantly greater odds of having a caesarean delivery than those with less educated husbands, particularly in the Barisal (OR: 4.48; 95% CI: 2.92, 6.87), Chattogram (OR: 2.69; 95% CI: 1.97, 3.68), Dhaka (OR: 2.04; 95% CI: 1.55, 2.69), Khulna (OR: 2.00; 95% CI: 1.38, 2.89), Rajshahi (OR: 1.93; 95% CI: 1.35, 2.75), Rangpur (OR: 2.46; 95% CI:1.62, 3.74) and Sylhet (OR: 1.76; 95% CI: 1.19, 2.60) divisions. In contrast, educated women also had a higher chance of having a caesarean delivery, ranging from 1.71 in the Rangpur division to 2.93 in the Sylhet division, compared to women with primary or no formal education. Women from households with higher wealth status had significantly higher odds (adjusted odds ratio ranging from 1.39 in Rajshahi division to 2.95 in Sylhet divisions) of having a caesarean delivery than women from low wealth status households across all divisions. However, except the Rangpur division, overweight or obese women had a higher chance of having a caesarean delivery than the women with normal weight in Barisal (OR: 1.85; 95% CI: 1.35, 2.54), Chattogram (OR: 1.97; 95% CI: 1.58, 2.47), Dhaka (OR: 2.33; 95% CI: 1.91, 2.85), Khulna (OR: 1.80; 95% CI:1.37, 2.36), Rajshahi (OR: 1.94; 95% CI: 1.45, 2.60) and Sylhet (OR: 2.50; 95% CI: 1.85, 3.38), respectively. The effects of the factors on caesarean deliveries with the interaction effects of survey time and division are also presented in Table 3.

**Table 3:** Region-specific determinants of caesarean delivery among the ever-married women aged 15-49 years in Bangladesh.

Barisal	Chattogram	Dhaka	Khulna	Rajshahi	Rangpur	Sylhet	Pooled Data	Survey & division interaction
Model-1	Model-2	Model-3	Model-4	Model-5	Model-6	Model-7	Model-8	Model-9
Characteristic	OR (95% CI)^1^	OR (95% CI)^1^	OR (95% CI)^1^	OR (95% CI)^1^	OR (95% CI)^1^	OR (95% CI)^1^	OR (95% CI)^1^	OR (95% CI)^1^	OR (95% CI)^1^
**Women’s current age (in years)**									
15–19 years	Reference	Reference	Reference	Reference	Reference	Reference	Reference	Reference	Reference
20–34 years	1.11 (0.72, 1.71)	1.39 (1.01, 1.91) *	0.90 (0.69, 1.18)	1.15 (0.82, 1.62)	1.38 (0.95, 1.98)	1.10 (0.74, 1.65)	2.13 (1.26, 3.60) **	1.20 (1.05, 1.37) **	1.19 (1.05, 1.36) **
35–49 years	1.70 (0.80, 3.60)	1.28 (0.72, 2.29)	1.04 (0.65, 1.67)	1.95 (1.03, 3.72) *	2.84 (1.45, 5.58) **	2.97 (1.50, 5.91) **	3.01 (1.42, 6.37) **	1.78 (1.41, 2.23) ***	1.72 (1.37, 2.17) ***
**Women’s educational status**									
No formal education or primary	Reference	Reference	Reference	Reference	Reference	Reference	Reference	Reference	Reference
Secondary	1.19 (0.84, 1.69)	1.58 (1.20, 2.07) **	1.67 (1.37, 2.04) ***	1.34 (1.00, 1.79)	1.44 (1.06, 1.95) *	1.07 (0.76, 1.49)	1.55 (1.15, 2.09) **	1.48 (1.33, 1.64) ***	1.47 (1.32, 1.63) ***
Higher	1.94 (1.21, 3.11) **	2.12 (1.45, 3.09) ***	1.88 (1.38, 2.56) ***	2.63 (1.68, 4.11) ***	2.68 (1.71, 4.19) ***	1.71 (1.06, 2.75) *	2.93 (1.85, 4.66) ***	2.29 (1.96, 2.66) ***	2.27 (1.94, 2.64) ***
**Women’s age at first birth**									
< 18 years	Reference	Reference	Reference	Reference	Reference	Reference	Reference	Reference	Reference
≥ 18 years	1.19 (0.86, 1.64)	1.10 (0.87, 1.39)	1.36 (1.11, 1.67) **	1.28 (1.00, 1.65)	1.19 (0.90, 1.57)	1.50 (1.10, 2.05) *	1.14 (0.81, 1.60)	1.18 (1.07, 1.30) ***	1.24 (1.12, 1.36) ***
**Husband’s educational status**									
No formal education or primary	Reference	Reference	Reference	Reference	Reference	Reference	Reference	Reference	Reference
Secondary	2.21 (1.59, 3.08) ***	1.50 (1.18, 1.91) ***	1.25 (1.02, 1.53) *	1.21 (0.94, 1.57)	1.21 (0.92, 1.61)	1.62 (1.18, 2.20) **	1.25 (0.93, 1.67)	1.39 (1.26, 1.53) ***	1.37 (1.24, 1.51) ***
Higher	4.48 (2.92, 6.87) ***	2.69 (1.97, 3.68) ***	2.04 (1.55, 2.69) ***	2.00 (1.38, 2.89) ***	1.93 (1.35, 2.75) ***	2.46 (1.62, 3.74) ***	1.76 (1.19, 2.60) **	2.29 (2.01, 2.61) ***	2.22 (1.95, 2.53) ***
**Husband’s occupation**									
Agriculture									
Non-agriculture	0.92 (0.57, 1.48)	1.66 (1.10, 2.51) *	1.57 (1.18, 2.09) **	0.83 (0.63, 1.10)	1.00 (0.76, 1.33)	1.36 (1.00, 1.86) *	1.15 (0.80, 1.66)	1.10 (0.98, 1.24)	1.15 (1.02, 1.30) *
Others	1.07 (0.43, 2.68)	1.10 (0.57, 2.12)	1.83 (1.07, 3.13) *	1.02 (0.50, 2.08)	0.34 (0.11, 1.00)	1.52 (0.62, 3.73)	1.13 (0.59, 2.19)	1.02 (0.79, 1.33)	1.10 (0.85, 1.42)
**Place of residence**									
Rural	Reference	Reference	Reference	Reference	Reference	Reference	Reference	Reference	Reference
Urban	1.72 (1.28, 2.31) ***	0.93 (0.74, 1.16)	1.15 (0.95, 1.40)	0.92 (0.72, 1.18)	1.39 (1.06, 1.82) *	1.26 (0.93, 1.70)	0.90 (0.68, 1.19)	1.14 (1.04, 1.26) **	1.12 (1.02, 1.22) *
**Religion**									
Others	Reference	Reference	Reference	Reference	Reference	Reference	Reference	Reference	Reference
Muslim	0.82 (0.50, 1.34)	0.76 (0.54, 1.07)	0.42 (0.29, 0.61) ***	1.28 (0.91, 1.80)	0.81 (0.50, 1.31)	0.76 (0.53, 1.07)	0.81 (0.57, 1.15)	0.81 (0.70, 0.93) **	0.78 (0.68, 0.90) ***
**Household wealth index**									
Poor	Reference	Reference	Reference	Reference	Reference	Reference	Reference	Reference	Reference
Middle	1.27 (0.89, 1.83)	1.37 (0.99, 1.89)	1.29 (0.99, 1.66)	1.82 (1.33, 2.47) ***	0.98 (0.71, 1.36)	1.51 (1.06, 2.14) *	1.34 (0.87, 2.07)	1.35 (1.20, 1.52) ***	1.38 (1.23, 1.56) ***
Rich	1.92 (1.33, 2.78) ***	2.01 (1.48, 2.72) ***	1.76 (1.37, 2.25) ***	2.22 (1.64, 3.01) ***	1.39 (0.99, 1.95)	2.07 (1.44, 2.98) ***	2.95 (2.03, 4.27) ***	1.94 (1.73, 2.18) ***	2.03 (1.80, 2.28) ***
**Exposed to mass media**									
No	Reference	Reference	Reference	Reference	Reference	Reference	Reference	Reference	Reference
Yes	1.56 (1.15, 2.11) **	1.55 (1.21, 1.99) ***	1.71 (1.39, 2.12) ***	1.24 (0.94, 1.62)	1.25 (0.92, 1.68)	1.23 (0.91, 1.66)	1.54 (1.14, 2.07) **	1.55 (1.41, 1.72) ***	1.47 (1.33, 1.62) ***
**Birth Order**									
3+	Reference	Reference	Reference	Reference	Reference	Reference	Reference	Reference	Reference
1	1.85 (1.20, 2.86) **	1.82 (1.36, 2.43) ***	1.72 (1.32, 2.23) ***	2.01 (1.36, 2.97) ***	3.05 (2.04, 4.56) ***	1.83 (1.20, 2.79) **	2.65 (1.87, 3.76) ***	2.12 (1.86, 2.41) ***	2.02 (1.77, 2.29) ***
2	1.57 (1.07, 2.32) *	1.04 (0.80, 1.37)	1.26 (0.99, 1.59)	1.62 (1.16, 2.27) **	1.63 (1.14, 2.33) **	1.66 (1.14, 2.42) **	1.98 (1.42, 2.75) ***	1.51 (1.35, 1.70) ***	1.45 (1.29, 1.62) ***
**Maternal body mass index (BMI < 18.5)**									
Normal	Reference	Reference	Reference	Reference	Reference	Reference	Reference	Reference	Reference
Underweight	0.64 (0.44, 0.94) *	0.97 (0.73, 1.29)	0.74 (0.59, 0.94) *	0.76 (0.56, 1.02)	0.76 (0.56, 1.03)	0.82 (0.59, 1.14)	0.55 (0.39, 0.77) ***	0.74 (0.66, 0.83) ***	0.74 (0.66, 0.83) ***
Overweight or Obese	1.85 (1.35, 2.54) ***	1.97 (1.58, 2.47) ***	2.33 (1.91, 2.85) ***	1.80 (1.37, 2.36) ***	1.94 (1.45, 2.60) ***	1.28 (0.92, 1.78)	2.50 (1.85, 3.38) ***	1.98 (1.79, 2.18) ***	1.96 (1.78, 2.17) ***
**Pregnancy complications**									
No	Reference	Reference	Reference	Reference	Reference	Reference	Reference	Reference	Reference
Yes	1.57 (1.21, 2.04) ***	1.43 (1.18, 1.75) ***	1.66 (1.41, 1.96) ***	1.04 (0.83, 1.30)	1.54 (1.22, 1.95) ***	1.57 (1.22, 2.01) ***	2.07 (1.61, 2.66) ***	1.53 (1.41, 1.66) ***	1.52 (1.40, 1.65) ***
**Survey Year**									
2011	Reference	Reference	Reference	Reference	Reference	Reference	Reference	Reference	Reference
2014	1.70 (1.17, 2.49) **	1.50 (1.15, 1.96) **	1.82 (1.40, 2.37) ***	1.68 (1.26, 2.24) ***	1.58 (1.15, 2.18) **	1.64 (1.14, 2.35) **	1.09 (0.77, 1.56)	1.59 (1.42, 1.79) ***	1.66 (1.16, 2.38) **
2017	2.56 (1.77, 3.70) ***	2.42 (1.86, 3.16) ***	2.28 (1.79, 2.89) ***	2.40 (1.80, 3.19) ***	2.59 (1.88, 3.58) ***	3.43 (2.35, 5.01) ***	2.41 (1.76, 3.30) ***	2.54 (2.28, 2.84) ***	2.41 (1.71, 3.39) ***
2022	6.12 (4.03, 9.30) ***	3.97 (2.92, 5.40) ***	4.27 (3.26, 5.58) ***	6.09 (4.25, 8.72) ***	4.04 (2.79, 5.86) ***	5.76 (3.78, 8.79) ***	2.16 (1.45, 3.20) ***	4.41 (3.89, 5.01) ***	5.47 (3.75, 7.97) ***
**Divisions**									
Barisal									
Chattogram									0.84 (0.60, 1.17)
Dhaka									1.41 (1.01, 1.98) *
Khulna									1.74 (1.24, 2.45) **
Rajshahi									1.43 (1.00, 2.04) *
Rangpur									0.89 (0.61, 1.30)
Sylhet									1.10 (0.77, 1.56)
Survey Year * Division									
2014 * Chattogram									0.89 (0.57, 1.38)
2017 * Chattogram									0.95 (0.62, 1.45)
2022 * Chattogram									0.65 (0.41, 1.04)
2014 * Dhaka									1.09 (0.70, 1.70)
2017 * Dhaka									0.94 (0.62, 1.43)
2022 * Dhaka									0.78 (0.49, 1.23)
2014 * Khulna									1.06 (0.67, 1.69)
2017 * Khulna									1.04 (0.67, 1.62)
2022 * Khulna									1.20 (0.72, 2.01)
2014 * Rajshahi									0.95 (0.59, 1.53)
2017 * Rajshahi									1.10 (0.69, 1.74)
2022 * Rajshahi									0.73 (0.44, 1.24)
2014 * Rangpur									0.96 (0.58, 1.59)
2017 * Rangpur									1.32 (0.82, 2.14)
2022 * Rangpur									1.00 (0.58, 1.69)
2014 * Sylhet									0.65 (0.40, 1.06)
2017 * Sylhet									0.93 (0.59, 1.46)
2022 * Sylhet									0.40 (0.24, 0.67) ***
AIC	1565.82	2836.46	3713.3	2139.94	1955.74	1782.38	1820.41	15821.63	15964.22
BIC	1699.07	2982.97	3862.45	2273.36	2089.7	1917.69	1961.28	16194.54	16150.68
Area under the curve	0.853	0.817	0.818	0.793	0.816	0.842	0.857	0.829	0.828
Intra-cluster correlation coefficient	0.012	0.055	0.013	0.020	0.025	0.057	0.006	0.025	0.046

^1^OR = adjusted Odds Ratio, CI = Confidence Interval, AIC = Akaike Information Criterion, BIC = Bayesian Information Criterion, ***p-value<0.001, ** p-value <0.01, * p-value <0.05;

## Discussion

This study analyzed the four most recent nationally representative pooled datasets from BDHS to explore region-specific variations and their determinants contributing to caesarean deliveries in Bangladesh. The rate of caesarean deliveries has drastically increased over time across all the regions of the country. This analysis revealed that educated couples have a higher contribution to the increased rate of caesarean deliveries in all regions. We also found that the caesarean deliveries were more prevalent among the women living in the wealthier households and those with a greater BMI. Additionally, the birth order of the children, survey time, and pregnancy complications were also found to be notable correlates of caesarean deliveries in all seven regions as well as in the pooled analysis.

Women with higher levels of education residing in the Sylhet, Rajshahi, and Khulna divisions had notably higher rates of caesarean deliveries compared to their counterparts. Rajshahi and Khulna divisions reported the second-highest rate of higher (more than secondary) education among the ever-married women [11]. Additionally, Khulna and Rajshahi regions also had higher rates of antenatal care utilization from medically trained personnel among pregnant women, while Sylhet had the highest fertility rates [28]. Prior fertility and desire for further children may result in more pregnancies, increasing the total number of caesarean deliveries [29]. Previous studies have also confirmed these region-specific disparities of caesarean delivery among educated women [30,31]. A possible explanation for this finding can be that women with greater levels of education are more likely to be of higher socioeconomic status, which influences their decision in favor of private delivery facilities and results in a higher number of caesarean deliveries [32]. Educated women tend to opt for caesarean sections due to perceived lower pain levels, the belief in increased safety, minimal disruptions with daily tasks, and a perception of greater social prestige compared to natural vaginal delivery [33]. Access to healthcare facilities, exposure to health information, and improved health-seeking behavior are all correlated with education levels [34]. It is likely that educated mothers comprehend both the pros and cons of caesarean deliveries, which enables them to choose this procedure [24]. To mitigate the long-term consequences of rising caesarean section rates, policymakers ought to notify and encourage educated women by highlighting both the advantages and potential risks of caesarean deliveries.

Our findings indicate that, in all the regions, the husband’s educational status was also an important predictor of caesarean deliveries. Women who live with highly educated husbands were more likely to undergo caesarean deliveries, a pattern observed consistently across all geographic regions, with the lowest odds recorded in the Sylhet division. Sylhet had the lowest proportion of husbands with formal education [11]. Limited education and low standard of living among husbands often hinder access to healthcare, which, in turn, affects maternal healthcare practices [25,35]. Prior studies have also found that partners with higher educational backgrounds were associated with caesarean deliveries [23]. According to a meta-analysis study on 36 Sub-Saharan African DHS data, the husband’s level of education was an important driver in the rising number of caesarean deliveries [36]. Meanwhile, educated husbands with better financial stability are often more knowledgeable about healthcare, and may play a more active role in decision-making regarding birth, which might influence the couple’s belief that having a caesarean delivery is safer and less disruptive to their workload and free time [35,36]. The policymakers could introduce culturally and socially tailored interventions that actively engage educated husbands to improve awareness and promote appropriate health behaviors, thereby discouraging unnecessary or elective cesarean deliveries.

This study identified a clear regional variation in maternal BMI and caesarean delivery. As expected, overweight and obesity were most prevalent among women in Dhaka, Khulna, and Rajshahi, who also reported higher rates of caesarean delivery. In contrast, regions such as Sylhet and Rangpur, despite showing moderate levels of overweight or obesity, appeared to experience lower rates of caesarean delivery. The Rajshahi, Dhaka, and Khulna divisions emerged as prominent hotspots for caesarean section deliveries [37]. Dhaka and Khulna had the highest prevalence of overweight and obesity in urban areas, while Rajshahi and Chittagong exhibited the highest rates in rural areas [38]. However, a prior study found that the maternal double burden of overweight and short stature increased the odds of caesarean delivery in South Asia [39]. Overweight mothers face potential complications such as gestational diabetes, miscarriage, labor induction, anesthetic complications, preeclampsia, fetal distress, and wound infections [40]. Excess weight can also make it difficult for the baby to pass through the birth canal, leading healthcare providers to recommend a caesarean delivery for the safety of overweight women [41,42]. Given the regional differences in caesarean delivery rates related to the severity of overweight or obesity among women, healthcare providers should offer personalized counseling to address specific health concerns. This should involve guidance on preventing additional weight gain, managing associated complications, encouraging vaginal delivery when possible, and promoting healthier overall outcomes.

According to this study, women who were exposed to media were more likely to have greater caesarean delivery rates than reference groups in Dhaka, Barisal, Chattogram, and Sylhet divisions. Although the proportion of women watching television at least once a week was higher in the Rajshahi, Dhaka, and Khulna divisions, regular internet use was notably more prevalent in Chattogram, Dhaka, and Sylhet, respectively [11]. Women residing in regions with greater Internet access were more likely to undergo a caesarean delivery [43]. Women who often viewed media depictions of caesarean deliveries were more inclined to choose one regardless of whether it was medically necessary [44]. The perceived safety and convenience of caesarean delivery may be frequently highlighted in the media, which could raise demand [44]. To cut the caesarean delivery rate in each location, policymakers could authorize national and local media to air animated videos or advertisements highlighting the health and financial benefits of normal vaginal delivery in several local languages.

The findings of this study indicate the need to examine the increasing prevalence of caesarean sections across various geographical areas. The study also found that the couples with higher educational attainment had a higher rate of caesarean births, particularly in Dhaka and Khulna divisions. Medically indicated caesarean deliveries are often performed in response to obstetric complications, when vaginal delivery might endanger the health mother or newborn health [45]. However, the long-term consequences of repeated caesarean deliveries can put mothers at risk for surgical complications, aberrant placentation in later pregnancies, and prolonged recovery periods [46]. For neonates, caesarean delivery has been linked to increased rates of respiratory difficulties, altered gut microbiome colonization, and potential long-term concerns such as asthma, allergies, and obesity [47].

The policymakers should focus on the provision of adequate maternal and child health services, considering the geographical variation, and make appropriate health facilities available by strengthening existing policies to reduce the public-private disparity in the provision of caesarean deliveries. Public health facilities must be upgraded to prevent economically and socially disadvantaged communities from incurring unmanageable health cost.

### Strengths and limitations

The survey inquired about the most recent live births that took place within the three years before the study among the mothers. The accessibility, cost, and quality of delivery services vary by region, and the decision-making role of women is likely to influence delivery practices. Due to the lack of access to medical records in this household-based survey, this study was unable to fully explain the effects of those factors. Since BDHS data are cross-sectional, a causal relationship between covariates cannot be inferred. An advantage of this study is its comprehensive consideration of numerous confounding variables and its extensive examination of socio-demographic aspects in different regional areas, which may impact on caesarean deliveries.

## Conclusion

The study findings indicate that factors such as level of education, socioeconomic status, availability of healthcare services, and regional disparities can impact the frequency of caesarean deliveries. Considering geographical variations to ensure the provision of adequate maternal and child health services, the government should prioritize educating mothers and families about the benefits of normal delivery, enforce robust regulations, and implement strict oversight of healthcare services. This should include the use of hospital electronic medical records, data management systems, and standardized prescription protocols. The policymakers should take proactive steps based on this evidence. One potential intervention area could be the introduction of a centralized or local pregnancy registration system, which may help improve monitoring and access to maternal health services. Further exploration of the feasibility and implementation of such a system would be valuable.

## Supporting information

S1 Data Dataset: This supporting material included the dataset used in the study. (RAR)

S2 File This supporting material included the Stata data management syntax used in the study. (RAR)

S3 File This supporting material included the R analysis script used in the study. (RAR)
